# Mission-oriented determination of regional weapon maintenance demand in simulation

**DOI:** 10.1038/s41598-023-49769-9

**Published:** 2024-01-02

**Authors:** Hu Zhi-gang, Lou Jing-jun, Zeng bin

**Affiliations:** 1 Department of Management Engineering and Equipment Economics, Navy University of Engineering, Wuhan, 430033 China; 2 College of Naval Architecture and Ocean, Naval University of Engineering, Wuhan, 430033 China

**Keywords:** Energy science and technology, Engineering, Mathematics and computing

## Abstract

Determining maintenance demand ahead of mission is crucial to practical weapon maintenance, particularly to regional warship weapon maintenance. Attention is paid only to reliability, and the nature of mission or the consequence of damage is ignored while determining the regional warship weapon maintenance demand. For this reason, a method for determining regional maintenance demand based on simulation is put forward in this paper. Regional weapon maintenance system is first analyzed to build a mission-oriented maintenance demand model with the concept of mission-induced failure. Subsequently, the Anylogic platform is employed because of its advantages including agent modeling simulation and visualized process display. Four types of agent are designed for the regional maintenance system. The process of determining maintenance demand based on simulation is established on this basis. An example is eventually taken to calculate and verify the universality and effectiveness of the simulation model.

## Introduction

Maintenance demand is a basic input of a maintenance system. After determining maintenance demand, it is possible to allocate maintenance missions and dispatch maintenance resources. Therefore, the operation of the maintenance system is directly affected by the accurate determination of maintenance demand. Presently, maintenance demand is mainly determined from two approaches. In the development phase, maintenance demand is analyzed and determined by failure mode and effect analysis (FMEA), reliability centered maintenance analysis (RCMA), level of repair analysis (LORA), and operation and maintenance task analysis. It tends to allocate maintenance resources reasonably. In the application phase, empirical analysis is carried out to determine maintenance demand for dynamic dispatch of maintenance resources. According to the full lifecycle theory, static allocation is implemented prior to dynamic dispatch. Nevertheless, practical maintenance is noticeably characterized by missions in stages. At this time, maintenance demand is directed by the mission of combat training. It is therefore urgent to explore how to determine maintenance demand in a mission-oriented way. Maintenance resources are particularly addressed in this study on maintenance demand.

The mission-oriented determination of maintenance demand follows the basic idea of two phases, that is, development and application. Meanwhile, we take into account the maintenance demand in combat. Some studies have been carried out with regard to the determination of maintenance demand based on combat mission. In reference^1^, the synthetic military maintenance system was analyzed in terms of object, resource, and strategy to build a basic model for calculating the demand for maintenance personnel and spare parts. It was verified that the Monte Carlo simulation is feasible to the gradual synthetic calculation of the demand for maintenance personnel and spare parts. In reference^2^, it was claimed that maintenance resource demand must be determined with structured and unstructured aspects, so that a semi-structured prediction model was constructed for this purpose. In references^3–5^, maintenance personnel demand was predicted by quantifying the maintenance mission, determining the minimum maintenance unit, and employing the queuing theory. Considering the characteristics of maintenance system, a model was constructed with the mission, base, and weapon layers of the weapon maintenance system in reference^6^. Borrowing the multi-agent method, the model addressed two aspects, namely, structure and process, and took into account time and constraints in the maintenance mission. It was used to simulate the maintenance activities for typical missions. In reference^7^, it was argued that the key node identification technique for maintenance system was of great significance. It was pointed out that the importance of maintenance node can be measured by two capabilities, that is, connection within a certain spatial range and mission-demand-oriented global transportation. The comparison of indicators proved the good performance and stability of the proposed technique. Considering the characteristics of combat mission, reference^8^ presented the prediction based on the spare parts maintenance probability by classifying damages in a combat mission into technical damage and strike damage. On the basis of reference^9^, the maintenance demand was predicted with combat countermeasures, and the analytic hierarchy method was adopted to determine the contribution. The contribution was used to assign the maintenance probability. A spare parts prediction model was built for technical damage by employing the exponential distribution, Weibull distribution, and normal distribution. Because of fire combat, Bernoulli distribution was employed to build a spare parts prediction model for strike damage to weapon. Considering the simulation of maintenance activities, reference^10^ further took damaged weapon, enemy’s situation and commands as the inputs to direct the temporal and spatial distribution of damaged weapons, damaged parts, and repairmen in the operational plan. Thus simulation results were used to assess and predict maintenance demand, and further verify the advantages of the simulation prediction system. In reference^9^, it was pointed out that empirical analysis depended on subjective experience in the demand prediction of spare parts. An analytic model was based on the known service life distribution of spare parts, so that computer-aided simulation was more reliable. It was further pointed out that spare parts demand prediction deviated dramatically from the operational readiness requirement. A spare parts demand prediction model was therefore constructed on the basis of spare parts maintenance probability, but it was affected by the assignment of spare parts maintenance probability. These methods were compared in reference^11^. Based on their advantages and disadvantages, and considering the complexity and uncertainty of combat, a method was proposed by combining case-based reasoning and combat simulation.

Based on the above literatures, it is found that the maintenance demand prediction changed in two aspects, that is, gradual expansion from maintenance system to combat mission, and from maintenance system model to maintenance simulation model. On the whole, the overall characteristics of combat mission are taken into account, but specific characteristics are not analyzed. Therefore, the limitation of the analytic model is more noticeable with the expansion of scale. However, the simulation model becomes more and more advantageous. For this reason, the basis has been made for the mission-oriented determination of maintenance demand in the existing studies, but there are still three shortcomings: (1) reliability or combat counterwork is taken into account separately; (2) analytic model is favored among the methods, and there is not a simulation model for general conditions; (3) a few studies focus on strike damage (reference^8^), and only on how the demand is affected by the degree of combat, but how strike damage affects maintenance demand is not further explored.

In the mission-oriented determination of maintenance demand, the basic question is to find out the weapon maintenance resources needed for the weapon system to fulfill the assigned combat mission, so as to provide the inputs for subsequent allocation to maintenance missions and dispatching of maintenance resources. The contributions of this paper is: (1) put forth the concept of mission-induced failure. Mission-induced failure refers to a state in which weapon fails to function as specified in a mission, including technical failure and damage failure. Mission-induced analysis provides the basis for the analysis of maintenance demand in a mission. (2) Build a general simulation model. Empirical analysis and analytic model are evidently obstructed by structure of maintenance system and duration of calculation. Meanwhile, a simulation model must be applicable to both structure and system parameters. Its results are more reliable since they are gathered after long-time simulation. The simulation model is also more widely applicable since it takes into account the elements of the entire system including performance indicators, mission structure, weapon structure, resource constraint, and correlation.

In this paper, warship weapon is studied for mission success rate by considering the factors affecting mission-induced failure and the priority of mission weapon. A model for determining maintenance demand is established for simulation design based on multiple agents and Anylogic platform. The maintenance demand calculation process is determined and verified in a case study.

## Establishing a model

### Framework of the model

A mission-oriented maintenance demand model involves the objective and elements of the model and the relationship between such elements. In this paper, mission success rate of weapon (system) is taken as the objective of the model. Total mission success rate is an aggregate of mission success rates at all mission profiles. The mission success rate of each unit is determined on the basis of the mission success rate of weapon (system). The elements of the model include mission-induced failure, mission structure, and weapon (system) priority. Mission-induced failure is classified into technical failure and damage failure. The number of technical failures is calculated by mission intensity and failure rate, while the number of damage failures is determined by mission level and damage probability. Mission structure depends on mission profile and “mission-weapon” relationship, while weapon (system) priority is quantified according to the mission structure. The maintenance demand is finally directed by the mission success rate, and calculated by integrating mission-induced failure, priority, maintenance constraint, and maintenance strategy.

**Figure 1:**
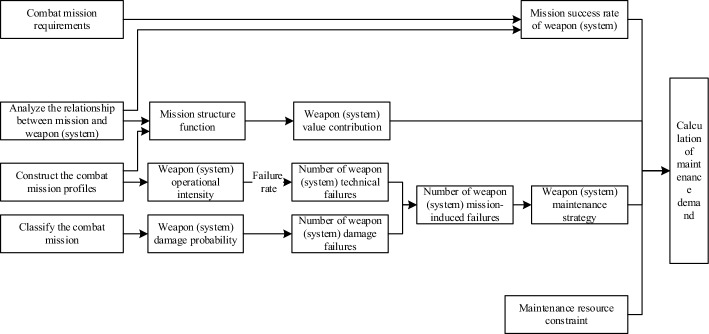
Basic framework of a maintenance demand model.

The basic framework for modeling support requirements is shown in Fig. 1.

### Problem description

Based on the basic framework of a maintenance demand model, the elements of the model are mathematically described as follows:

1. Unit combat mission ${U}_{i}$ is the smallest process unit of completing the combat function within a specified time period ${t}_{i}$, where $i$ stands for the number of missions;
2. Phased combat mission profile is a description of time sequence for the events and environments experienced within the time period of completing a specified phased mission. Assuming that a phased combat mission consists of N unit missions, which are executed serially, the unit mission sequence is $U=\left\{{U}_{1},{U}_{2},\dots ,{U}_{N}\right\}$;
3. Unit combat mission success rate ${S}_{{U}_{i}}\left({t}_{i}\right)$ means to judge whether a mission is successful based on the intactness of weapon (system) associated with the unit combat mission, that is, ${S}_{{U}_{i}}\left({t}_{i}\right)=\left\{\mathrm{1,0}\right\}$, where 1 means mission success and 0 stands for mission-induced failure.

When ${t}^{s}\left({U}_{i}\right)\le {t}_{1}^{c}\left({U}_{i}\right)-{t}_{2}^{c}\left({U}_{i}\right)$, ${S}_{{U}_{i}}\left({t}_{i}\right)=1$. When ${t}^{s}\left({U}_{i}\right)>\left({U}_{i}\right)-{t}_{2}^{c}\left({U}_{i}\right)$, ${S}_{{U}_{i}}\left({t}_{i}\right)=0$. Moreover, ${t}_{1}^{c}\left({U}_{i}\right)$ stands for the time needed by a unit combat mission; ${t}_{2}^{c}\left({U}_{i}\right)$ represents the duration of a unit combat mission; and ${t}^{s}\left({U}_{i}\right)$ indicates the time of maintenance.

4. Weapon availability ${r}_{{E}_{j}}$ means whether weapon ${E}_{j}$ is available. Availability is divided into technical state and service state. Technical state includes healthy, in repair, and to be repaired. Service state includes in service and standby. In this case, *j* = 1…*J*, where *J* stands for the number of equipments.
5. The time between failures ${BFT}_{{E}_{j}}$ of equipment ${e}_{k}$ is subject to Weibull distribution $\frac{{m_{{e_{k} }} }}{{\mu _{{e_{k} }} }}t^{{(m_{{e_{k} }}  - 1)}} e^{{ - \frac{{tm_{{e_{k} }} }}{{\mu _{{e_{k} }} }}}}$, where $k=1\dots K$, and $K$ is the number of equipments.
6. The maintenance time ${MT}_{{E}_{j}}$ of equipment ${e}_{k}$ is subject to normal distribution $\frac{1}{\sigma \sqrt{2\pi }}{e}^{-{(t-{\theta }_{{e}_{k}})}^{2}/(2{{\sigma }_{{e}_{k}}}^{2})}$.
7. Combat mission counterwork degree is denoted by ${CD}_{m}$, $m=\left\{\mathrm{1,2},3\right\}$, where 1 indicates the intense counterwork in a combat mission with ${CD}_{1}=0.3$; 2 represents the highly intense counterwork in a combat mission with ${CD}_{1}=0.5$; and 3 stands for the extremely intense counterwork in a combat mission with ${CD}_{1}=0.8$.
8. Value contribution ${v}_{k}$ of equipment ${e}_{k}$ means the importance of equipment in a unit combat mission. It is determined by combat mission structure and weapon structure. The mission structure function is $\mathrm{\varphi }=\left({e}_{1},{e}_{2},\dots {e}_{K}\right)$, and the weapon structure function is $\mathrm{\varnothing }=\left({e}_{1},{e}_{2},\dots {e}_{K}\right)$, so that ${v}_{k}={f}_{{e}_{k}}(\mathrm{\varphi },\mathrm{\varnothing })$.
9. Damage probability ${p}_{k}$ of equipment ${e}_{k}$ represents how possible equipment is damaged in a unit combat mission. It is determined by combat counterwork degree, equipment value contribution, and random uniform distribution function, that is, ${p}_{k}=f({CD}_{m},{v}_{k},random())$.
10. Unit combat mission success rate ${R}_{{U}_{i}}\left({t}_{i}\right)$ is the probability of unit combat mission success, ${R}_{{U}_{i}}\left({t}_{i}\right)=\left[1-\left(1-{r}_{{E}_{j}}\right)\left(1-{e}^{-\theta {t}_{2}^{c}\left({U}_{i}\right)}\right)\right]{S}_{{U}_{i}}\left({t}_{i}\right)$, where $\theta$ is the combat intensity coefficient, and $\left(1-{e}^{-\theta {t}_{2}^{c}\left({U}_{i}\right)}\right)$ represents the possibility of failure within the duration of mission. The longer duration of the mission, the higher possibility of failure.
11. Total combat mission success rate ${R}_{U}$ is a combination of unit combat mission success. If a unit mission fails, the total combat mission fails. Thus there is ${R}_{U}\left(T\right)=\prod_{i=1}^{n}{R}_{{U}_{i}}\left({t}_{i}\right)$, where $T=\sum_{i=1}^{n}{t}_{i}$.
12. There are $L$ types of maintenance resource ${\text{MR}}=\left\{{MR}_{1},{MR}_{2},\dots ,{MR}_{L}\right\}$, which are classified into personnel and spare parts in terms of nature, or occupied and consumed resources in terms of how to use.

### Maintenance strategy

1. In case of failure, equipment can be maintained by using original parts or replacing parts, which needs maintenance personnel and spare parts. Moreover, spare parts are classified into occupied and consumed parts. If maintenance is achieved with original parts, spare parts refer to the tools, which are occupied resources. If parts are replaced in the maintenance, spare parts are consumed resources.
2. Two-level maintenance mode is adopted. Maintenance relies mainly on warship crew, so that the application for and dispatch of personnel and spare parts are conducted within the specified mission support system.

## Simulation design

### Simulation framework

Simulation is an effective way to learn about the law of the real world, and its results are used to direct the real world for the intended purpose ^12^. After building the model for the mission-oriented determination of weapon maintenance demand, simulation can be achieved in various ways. As for a weapon maintenance system, it is necessary to calculate maintenance demand and understand the calculation process of maintenance demand. Particularly, unit missions, work state of weapon system, and consumption of maintenance resources are visualized to provide the visual data in multiple resolutions for maintenance demand decision and maintenance mission command. The Anylogic simulation platform utilizes state diagram and communication techniques. Moreover, it combines agent modeling and discrete event modeling ^13,14^ to rapidly and effectively simulate the elements of the weapon maintenance system including mission, weapon and resource. In this way, simulation can be fulfilled by virtue of visualized modeling.

**Figure 2:**
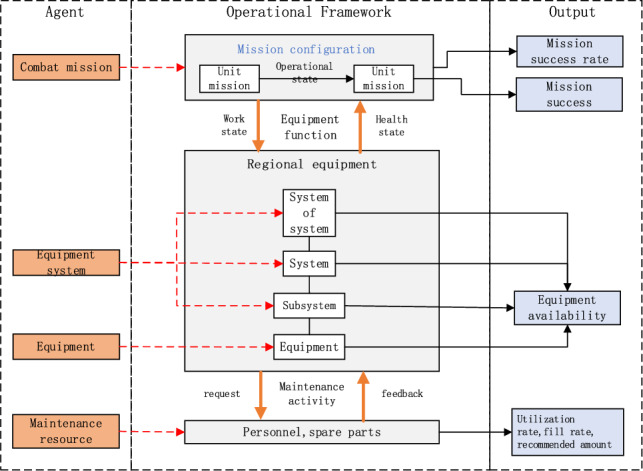
The framework of mission-oriented maintenance support agent simulation.

Considering the unit mission profile, weapon system structure, and maintenance demand plan, a maintenance system is abstracted into four agents including combat mission, weapon system, equipment, and maintenance resource. The interaction of these agents is analyzed. Combat mission success rate is broken down into “mission, system, equipment, resource” from top to bottom, so that it is reflected by the mission success of specific equipment. Moreover, efforts are made to coordinate “resource, equipment, system, mission” from bottom to top. Thus the mission success of specific equipment is comprehensively addressed and coordinated to achieve the desired combat mission success rate. Agents can be used to output such indicators as total combat mission success rate, unit combat mission success rate, weapon availability, maintenance resource utilization rate, maintenance resource fill rate, and recommended amount of maintenance resources. In this way, a mission-oriented maintenance demand agent simulation system is constructed with its operational framework illustrated in Fig. 2.

A weapon maintenance system is characterized by randomness. In order to stably determine maintenance demand, we follow the common process of combat mission, and use the practically reliable structure of weapon. It is also assumed that equipment failure is subject to Weibull distribution and equipment maintenance time follows normal distribution (assumption based on actual situation). Combat counterwork degree is divided into three levels, and damage randomness follows uniform distribution.

### Agent design

#### Combat mission agent design

**Figure 3:**
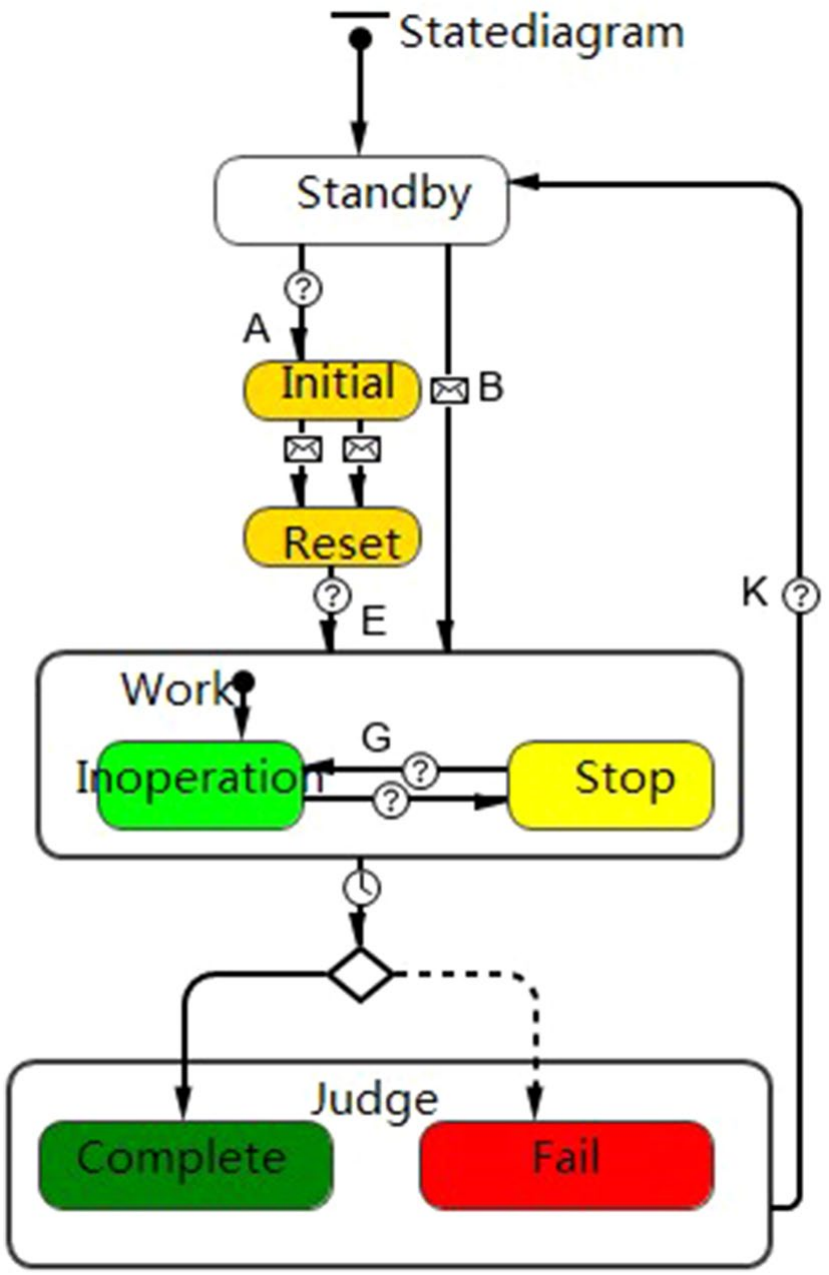
State diagram of unit mission agent.

Combat mission agent is a combination of unit mission agents. Unit mission agent design is divided into two aspects: (1) horizontal design. The work state is simulated for each unit mission. Information is transmitted via port between unit mission agents. (2) Vertical design. The influence of associated system agent states in the weapon system on unit mission agents is simulated. The design is as shown in Fig. 3.

#### Weapon system agent design

**Figure 4:**
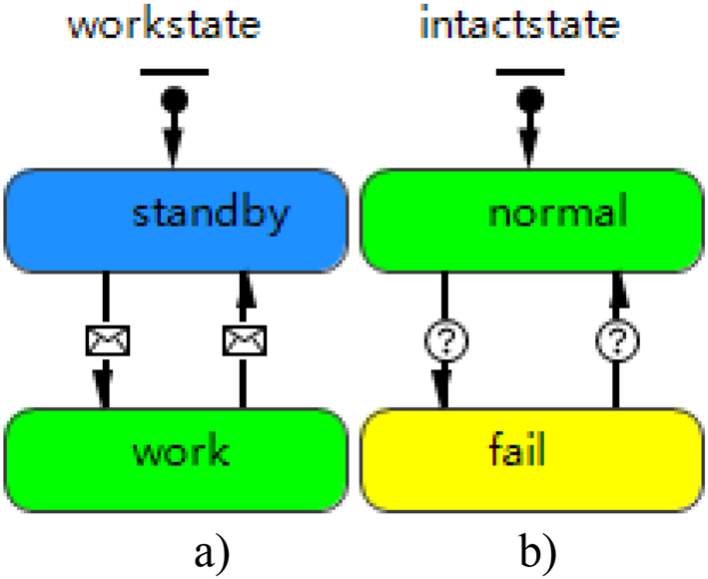
Weapon system agent: (**a**) work state diagram; (**b**) technical state diagram.

Weapon system agent design is compatible with the multilevel weapon system structure. It contains two parts: (1) design of work state and technical state. Work state and technical state are designed separately. Work state is responsible for transmitting state information to the components at lower level, so as to initiate the components. Technical state transmits state information to the components at upper level for confirming the restoration of normal state. (2) Design of information change and condition change. Information change transmits the global influence of work state, while condition change simulates the structure of weapon system. Weapon mission availability structure is used to simulate the influence of mission-induced failure on weapon system. The specific design is given in Fig. 4.

#### Weapon agent design

**Figure 5:**
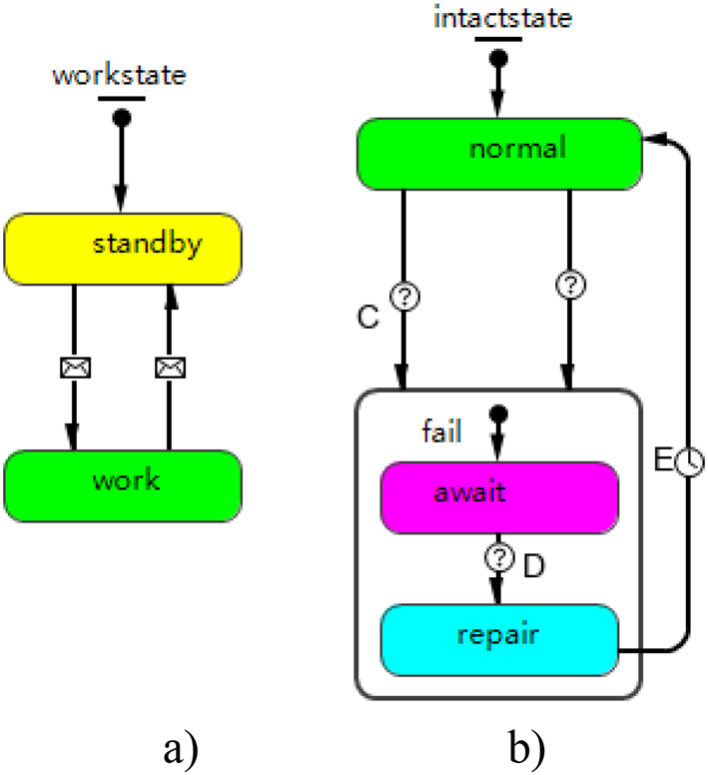
Equipment agent: (**a**) work state diagram; (**b**) technical state diagram.

Equipment agent design is further performed on the basis of the weapon system agent design. It is realized in two aspects: (1) Failure state, including “await for repair” and “in repair”, which are used to simulate the regularity of equipment failure and the time of repair. (2) Failure cause, including technical failure and damage failure. Technical failure results from wearing and aging of equipment, while damage failure is caused by the enemy’s fire strike. The design is as illustrated in Fig. 5.

#### Maintenance resource agent design

**Figure 6:**
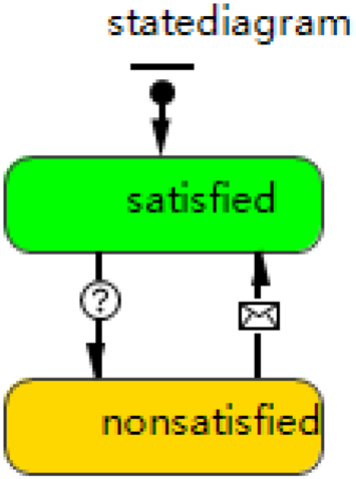
State diagram of maintenance resource agent.

The maintenance resource agent design takes into account the nature and type of resource, which is reflected in two aspects: (1) consumed and occupied resources. Personnel and tool kits are defined as occupied resource, while spare parts belong to consumed resource. (2) Resource allocation principles. Resources are allocated in order of request, which does not consider any other factor. The specific design is in Fig. 6.

### Calculation process

**Figure 7:**
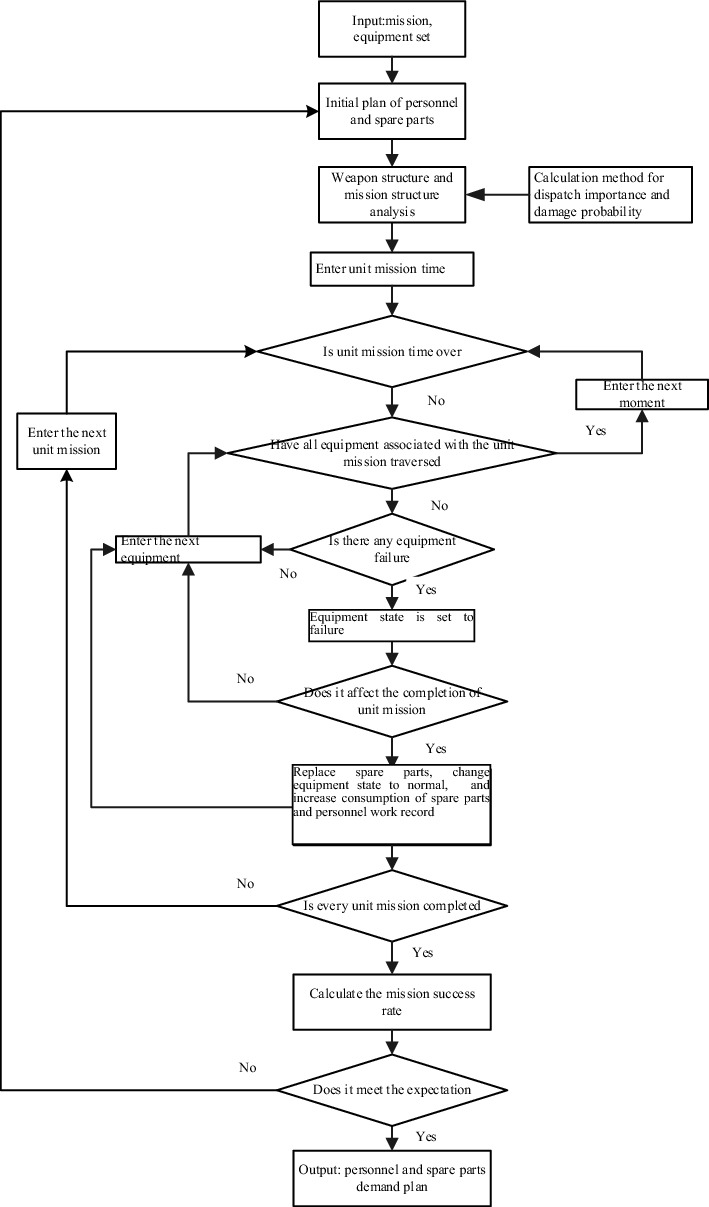
Simulation calculation process of mission-oriented weapon maintenance demand.

The mission-oriented weapon maintenance demand in the simulation is calculated in the following steps: (1) input the combat mission and weapon system; (2) define the initial plan of maintenance demand; (3) execute unit missions, and calculate maintenance demand and mission success rate; (4) if mission success rate is not as expected, adjust the initial plan of personnel and spare parts based on the recommended amount in the simulation results, and go back to Step (2); (5) if mission success rate is as desired, output the maintenance demand plan. The specific process is illustrated in Fig. 7.

## Case study

### Case background

**Figure 8:**
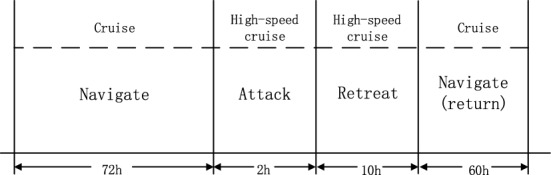
Process of a regional combat mission.

The process of a regional combat mission is given in Fig. 8.

**Figure 9:**
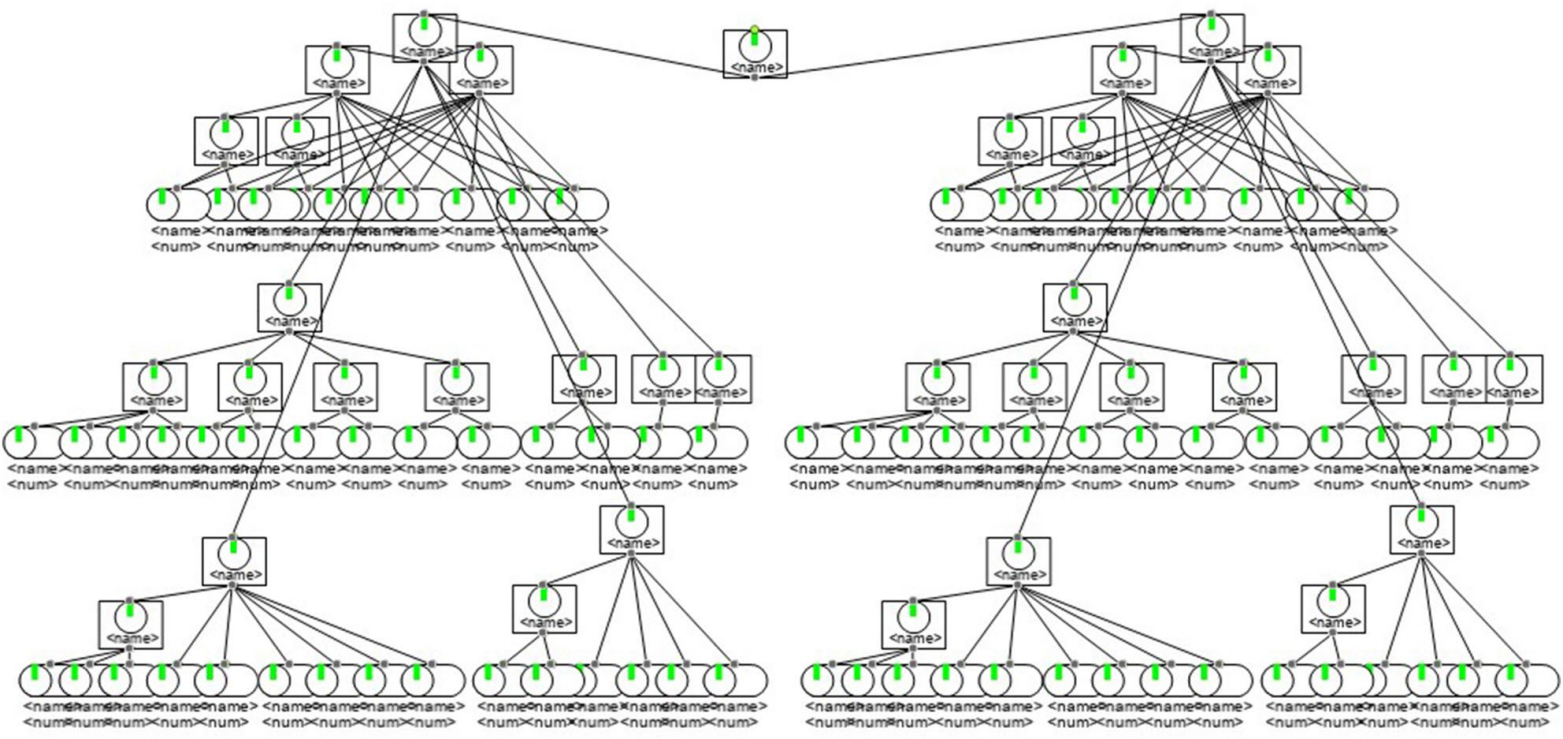
Weapon structure of two warships.

Two warships of the same type executed an attack mission in a formation. The parameters of its unit combat missions are given in Table 1. The technical parameters for warship weapon reliability are listed in Table 2. The weapon structure is illustrated in Fig. 9. The success rate of this combat mission is 0.82. The weapon maintenance demand of this combat mission is predicted in the subsequent sections.

**Table 1:** Parameters of unit combat missions.

Unit mission	Duration of mission	Allowed maintenance time	Combat counterwork degree
Navigate	72	6	1
Attack	2	0	3
Retreat	10	2	2
Return	60	4	1

**Table 2:** Technical parameters of warship weapon reliability.

Description	mtbf	mttr	Description	mtbf	mttr
1# diesel engine	2000	4	Platform compass 1#	2500	0.5
2# diesel engine	2000	4	Platform compass 2#	2500	0.5
3# diesel engine	2000	4	Electronic compass	3000	0.5
4# diesel engine	2000	4	GPS/GLONASS integrated satellite equipment	2400	0.5
1# gearbox	8000	4	Loran-C navigation receiver	2000	0.5
2# gearbox	8000	4	Ship’s log	2000	0.5
1# adjustable pitch propeller and shaft system	6000	4	Micro-logger	3000	0.5
1# adjustable pitch propeller and shaft system	6000	4	Sounder	2000	0.5
Surveillance	2000	0.5	Navigation console	2000	0.5
Auxiliary power unit	2000	2	Tracking radar	300	0.5
Accident distribution system	10,000	2	Opto-electronic tracker	420	0.5
Power distribution subsystem	10,000	2	Fire control equipment	360	0.5
1# main switchboard	10,000	8	Speed-measuring radar	400	0.5
2# main switchboard	10,000	8	Strapdown upright reference system	1500	0.5
Power station surveillance	10,000	2.5	XX naval gun	320	0.5
Local control box	10,000	2	Communication system	1400	2
1# diesel generator set	2800	8	Auxiliary system	1200	2
2# diesel generator set	2800	8	XX2 radar	500	0.5
3# diesel generator set	2800	8	Identification of friendly or foe (IFF)	600	0.5
4# diesel generator set	2800	8

### Calculated results and discussion

#### Calculated results

**Figure 10:**
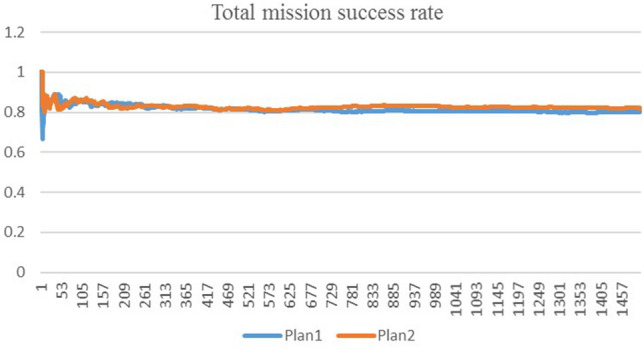
Calculated total mission success rate of two plans.

The simulation was implemented for 1500 times for the personnel and spare parts plan 1. The total mission success rate was calculated to be 0.80. After it was changed to the personnel and spare parts plan 2, the simulation was also implemented for 1500 times. The total mission success rate was calculated to be 0.82. The results are given in Fig. 10. According to the requirement for total mission success rate, it is determined that the maintenance support demand is based on plan 2 of Table 3.

**Table 3:** Personnel and spare parts plans (plan 1/plan 2).

Description	Synchronous capacitor	Sealing ring	Spacer	Bolt	Pressure sensor	Temperature sensor
Quantity	2/2	2/2	2/2	2/2	4/4	2/2
Description	Diode	Circuit breaker	Relay	Booster	Desuperheater	Pushbutton
Quantity	3/3	2/2	2/2	1/1	1/1	10/11
Description	Indicator lamp	Rectifier	Check valve	Fuse	Gear	Level sensor
Quantity	9/10	2/2	1/1	2/2	4/4	3/3
Description	Mechanical maintainer middle-level	Mechanical maintainer middle-level	Electrical maintainer middle-level	Electrical maintainer senior-level	Tool kit 1#	Tool kit 2#
Quantity	3/3	3/3	2/2	2/2	2/2	2/2

#### Discussion

During calculation, the analytic method faces the complexity of modeling and the difficulty in coping with the randomness of mission failure. Nevertheless, simulation offers a good way to cope with such complexity and randomness, so that it is adopted in this paper. As revealed in Fig. 10, the calculated results converge satisfactorily. The initial values fluctuate significantly at the beginning of calculation, but the total mission success rate becomes stable after simulation is carried out for 600 times. In the simulated calculation, different total mission success rates are obtained by adjusting the number of personnel and spare parts. Furthermore, two phenomena are noticed in the test. First, the total mission success rate is improved by increasing the number of personnel and spare parts before its saturation level, but it cannot be further improved after the saturation level. Second, the number of key personnel and spare parts exerts a more noticeable effect on the total mission success rate. The parameters of the model include unit combat mission parameters and warship weapon reliability technical parameters. These parameters are determined considering the characteristics of combat mission and actual weapon. In this paper, the parameters are simulated for a specific mission.

### Sensitivity analysis

This paper mainly focuses on how the mission success rate is affected by maintenance demand. However, mission success rate also depends on mission structure, failure regularity, combat counterwork, and demand plan. Maintenance demand must be therefore determined to not only calculate the mission success rate, but also arrange the environmental parameters for the mission, including mission structure, failure regularity, and counterwork degree. In the sensitivity analysis, it is necessary to analyze the sensitivity to environmental parameters and then the sensitivity to demand plan.

#### Sensitivity of total mission success rate to mission structure

**Figure 11:**
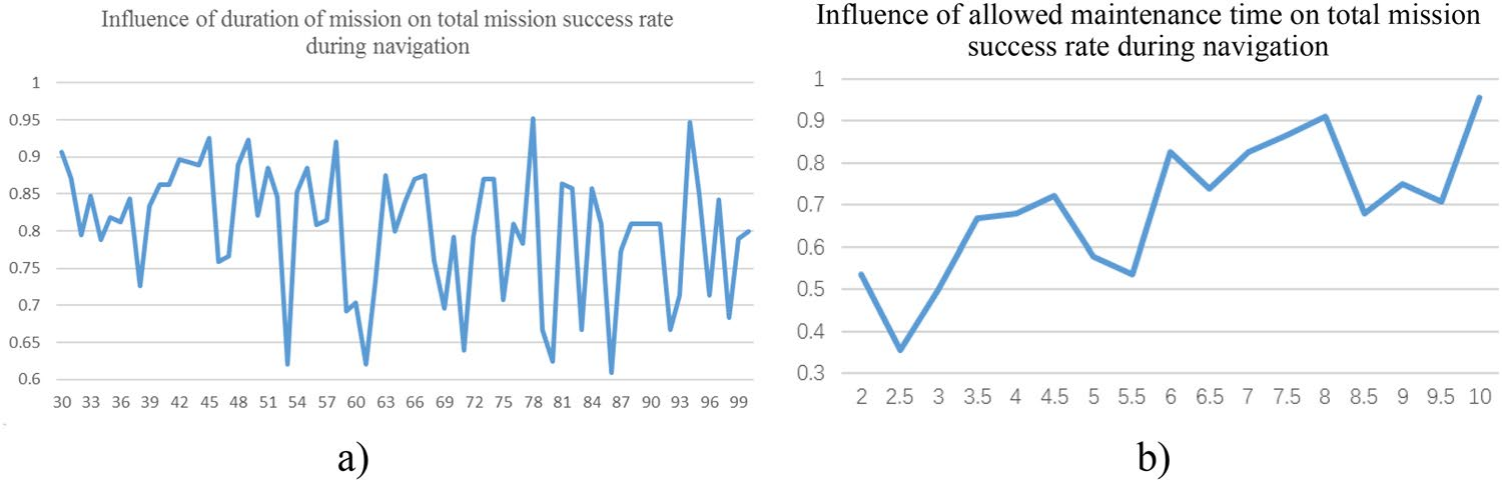
Sensitivity of total mission success rate to mission structure.

The success rate of a phased mission varies with the duration of the phased mission and the allowed phased maintenance time, so that it can affect total mission success rate. Taking the navigation phase as an example, the analysis results are illustrated in Fig. 11. When the duration of phased mission varies within a range of [30, 100], the total mission success rate fluctuates within a range of [0.6, 0.95]. In other words, the mission success rate does not increase with the duration of mission, but fluctuates within a certain range. Therefore, the duration of phased mission has a little influence on the total mission success rate. When the allowed maintenance time varies within a range of [2, 10], the total mission success rate increases with it. After all, the total mission success rate is slightly affected by the duration of mission, but dramatically by the maintenance time. Nevertheless, the allowed maintenance time is limited for a certain mission.

#### Sensitivity of total mission success rate to failure regularity

**Figure 12:**
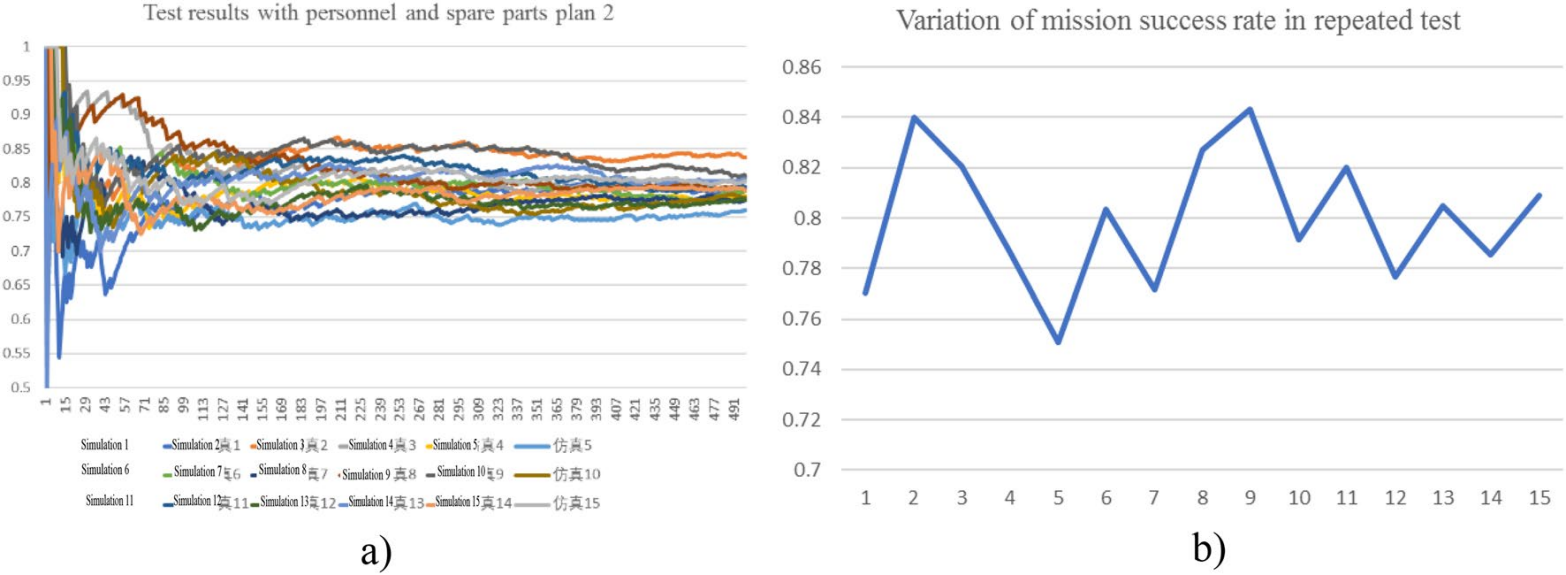
Sensitivity of total mission success rate to failure regularity.

The simulation was carried out for 500 times with the personnel and spare parts plan 2. In order to eliminate the influence of failure randomness error, the test was repeated for 15 times to obtain the results as illustrated in Fig. 12. Randomness affects mission success rate to some extent, but the mission success rate fluctuates within a range of [0.75, 0.84] and its average is 0.82. In other words, the influence of randomness on mission success rate is controllable. The calculated results after repeating the test with the personnel and spare parts plan 2 are reliable.

#### Sensitivity of mission success rate to combat counterwork.

**Figure 13:**
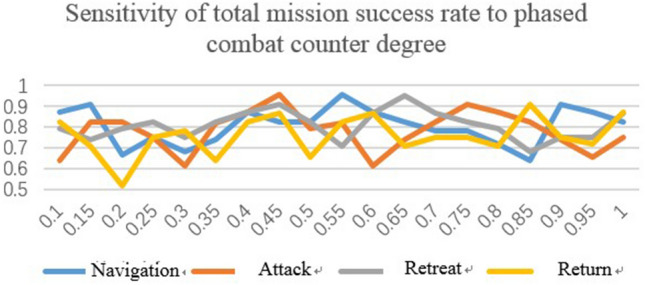
Sensitivity of mission success rate to combat counterwork.

Counterwork degree varies in phases. It is used to determine equipment damage probability. Subsequently, repair and maintenance can be arranged for damaged equipment. Simulation is carried out to calculate the influence of equipment availability and system availability on mission success rate. The sensitivity distribution is presented in Fig. 13. As shown in the figure, the mission duration in the phase of attack is only 2 h (which is 1/36, 1/5, and 1/30 of the mission duration of navigate, retreat, and return, respectively), implying that the combat level is highly sensitive to mission success rate.

#### Sensitivity of mission success rate to personnel and spare parts

**Figure 14:**
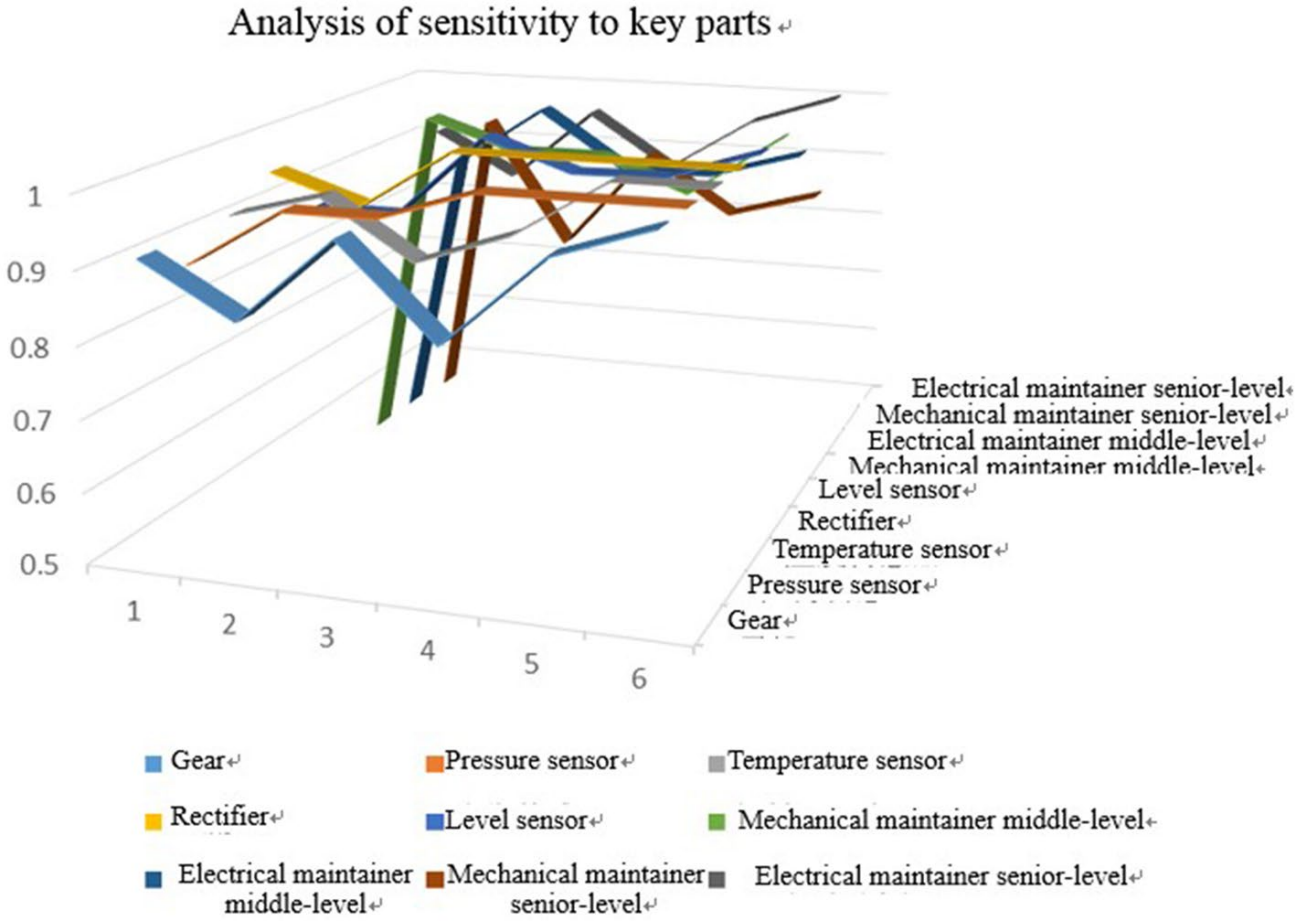
Sensitivity of mission success rate to personnel and spare parts.

When the environmental parameters of a mission are certain, mission success rate is mainly affected by the personnel and spare parts plan. The navigation phase is taken as an example to analyze the influence of personnel and spare parts on mission success rate. The distribution of sensitivity of mission success rate to some key parts is given in Fig. 14. Mission success rate is more sensitive to mechanical maintainer middle-level, electrical maintainer middle-level, and mechanical maintainer senior-level, but insensitive to other personnel. In the navigation phase, more attention should be paid to mechanical maintainer middle-level, electrical maintainer middle-level, and mechanical maintainer senior-level,

## Conclusion

With the general framework for determining the regional weapon maintenance demand, this paper proposes a simulation model based on multiple agents and Anylogic platform to effectively determine the regional weapon maintenance demand in a mission-oriented way. The innovation is reflected in the following aspects:

1. Based on the concept of mission failure, a mission-oriented maintenance demand analysis model is constructed to provide a scientific basis for the regional weapon maintenance demand analysis in a mission.
2. With the universal agent-based simulation model, a determination process of regional maintenance demand is established on the basis of simulation to provide a method for determining the regional weapon maintenance demand. Compared with analysis method, the proposed method is applicable to both the structure and system parameters, and its calculated results are more reliable.
3. The method for the mission-oriented prediction of regional maintenance demand provides the reliable prediction results. It provides a solid basis for further allocating regional maintenance and dispatching resources in a mission.

This paper mainly focuses on the determination of maintenance demand. In the case study, two ships with the same equipment were assigned to execute a simple mission. In the future, different types of equipment will be explored to execute complicated missions, so as to more systematically allocate maintenance demand and dispatch resources to missions.

### Supplementary Information

Supplementary Information.

## Data Availability

All data generated or analysed during this study are included in this published article [and its supplementary file].

## References

[1] Shuangchuan W, Xisheng J, Feng Li (2020). Demand determination and performance assessment of supporting resources for synthetic military equipment maintenance based on simulation. Acta Armamentarii.

[2] Shijian Z (2015). Semi-structural prediction method study on support resources demand of warship equipment. J. Nav. Univ. Eng..

[3] Qiu YL, Wang ZY, Zhang WJ (2011). Equipment support operation and maintenance manpower requirement forecasting model based on support activity flow. J. Acad. Equip. Command Technol..

[4] Luo MY, Liu T (2013). Research on the model of equipment maintenance personnel demand in the troops. J. Acad. Equip..

[5] Shi LH, Chen J, Ye ZQ (2019). Requirement analysis of POL equipment maintenance personnel based on queuing theory. Command Control Simul..

[6] Junsen L, Yining F, Yunan Z, Guanghan B, Junyong T (2023). A multi-agent modeling and assessment method for mission-oriented weapon maintenance system. J. Syst. Eng. Electron..

[7] Zong W, Huiliang S, Yongxiang X, Guanghan B, Yining F (2022). Analysis on key nodes in weapon maintenance system. J. Syst. Eng. Electron..

[8] Hui X, Guangyu L, Tielin L (2018). Spare parts support probability distribution and demand forecasting model based on combat mission. J. Acad. Armored Force Eng..

[9] Xiaochuang T, Linhan G, Boping X (2012). Demand prediction model for spare parts based on fill rate allocation. Acta Armamentarii.

[10] Zhang Haohua XS (2013). Study on maintenance support demand prediction at wartime based on simulation. J. Syst. Simul..

[11] Yaxin T, Rui F, Dong X (2015). Research on prediction method of army equipment maintenance requirement based on operation simulation case-reasoning. J. Syst. Simul..

[12] Yang H, Yifan X, Lv J, D’Aniello G (2020). An accelerated simulation approach for multistate system mission reliability and success probability under complex mission. Math. Probl. Eng..

[13] Chang P-C (2022). Simulation approaches for multi-state network reliability estimation: Practical applications. Simul. Model. Pract. Theory.

[14] Cao J, Guo Y, Zhang C (2021). Research on simulation and optimization of maintenance support mode and human resources auocation for complex equipment. J. Syst. Simul..

